# Influenza A (H1N1) in Victoria, Australia: A Community Case Series and Analysis of Household Transmission

**DOI:** 10.1371/journal.pone.0013702

**Published:** 2010-10-28

**Authors:** Clare Looker, Kylie Carville, Kristina Grant, Heath Kelly

**Affiliations:** Victorian Infectious Diseases Reference Laboratory, Melbourne, Australia; University of Georgia, United States of America

## Abstract

**Background:**

We characterise the clinical features and household transmission of pandemic influenza A (pH1N1) in community cases from Victoria, Australia in 2009.

**Methods:**

Questionnaires were used to collect information on epidemiological characteristics, illness features and co-morbidities of cases identified in the 2009 Victorian Influenza Sentinel Surveillance program.

**Results:**

The median age of 132 index cases was 21 years, of whom 54 (41%) were under 18 years old and 28 (21%) had medical co-morbidities. The median symptom duration was significantly shorter for children who received antivirals than in those who did not (p = 0.03). Assumed influenza transmission was observed in 63 (51%) households. Influenza-like illness (ILI) developed in 115 of 351 household contacts, a crude secondary attack rate of 33%. Increased ILI rates were seen in households with larger numbers of children but not larger numbers of adults. Multivariate analysis indicated contacts of cases with cough and diarrhoea, and contacts in quarantined households were significantly more likely to develop influenza-like symptoms.

**Conclusion:**

Most cases of pH1N1 in our study were mild with similar clinical characteristics to seasonal influenza. Illness and case features relating to virus excretion, age and household quarantine may have influenced secondary ILI rates within households.

## Introduction

The experience of pandemic influenza A (pH1N1) amongst hospitalised patients in Victoria [1] and overseas [2], [3], [4], [5], [6], [7] has been widely reported. Less information is available for community cases, despite it now being clear that the vast majority of cases were mild and occurred in non-hospitalised patients. [8], [9]

On 20^th^ May 2009, the Victorian Department of Health confirmed the first case of 2009 pH1N1 in the state, the second case in Australia. In total 3,089 cases and 26 deaths from pH1N1 were notified in Victoria in 2009. [10] Concern about clinical severity and community vulnerability during the initial phases of the H1N1 pandemic led to the implementation of a number of mitigation measures and significant social disruption. Confirmed cases were isolated, close contacts quarantined, and schools or classrooms in which there were confirmed cases closed. Such public health measures received widespread media coverage and caused significant community concern. [11], [12], [13], [14]

Quarantine measures also potentially altered the risk of infection to household contacts of a case. Recent work by Cauchemez found lower transmissibility of the 2009 pH1N1 virus in US households than in previous pandemics. [15] Previous studies of seasonal influenza have found the transmission of influenza in households can be influenced by age, family structure, circulating virus and exposure in the community. [16], [17] The patterns of transmission of pH1N1 in Australian households remains largely unexamined to date.

In this case series, we aimed to describe the epidemiological characteristics, clinical features and treatment of Victorian sentinel surveillance patients with laboratory-confirmed pH1N1. We also aimed to identify case and contact factors that may have impacted on the incidence of influenza-like illness (ILI) amongst household contacts of cases with confirmed pandemic influenza infection.

## Methods

### Victorian general practice sentinel surveillance network

Cases were identified through the Victorian General Practice Sentinel Surveillance scheme. [18] In 2009 this scheme comprised 87 metropolitan and rural general practitioners (GPs). Patients presenting to participating GPs with ILI were asked to consent to a combined nose and throat swab. The formal definition of ILI used in the surveillance program is based on the symptom triad of fever, cough and fatigue. This case definition has been found to have a positive predictive value for laboratory-confirmed influenza of between 23.3 and 59.7%. [19] On occasion participating GPs may swab patients in whom they suspect influenza despite the patient not having all three symptoms, for example, perhaps having rhinorrhoea instead of cough. We encouraged GPs to apply the same criteria for ILI in 2009 as they used in previous influenza seasons. GPs collected data on age, sex, symptoms and vaccination status of the patient. Recruitment for testing was at the discretion of the GP.

### Laboratory testing

Nose and throat swabs were sent to the Victorian Infectious Diseases Reference Laboratory (VIDRL), a World Health Organization National Influenza Centre. Testing for influenza A viruses involved extraction of RNA from nose/throat swabs. cDNA was derived by reverse transcription using random hexamers and amplified using fast real-time PCR incorporating primers and probes targeting the matrix gene of influenza A. Samples testing positive in the screening assay were confirmed as positive or negative for the pandemic virus in a second real-time PCR assay incorporating primers and probes specific for the haemagglutinin gene of that virus.

### Study recruitment

Eligible study participants were defined as all surveillance patients who had tested positive to pH1N1 between 1^st^ May (the start of the surveillance season) and 31^st^ August 2009 (when we commenced recruitment for this study) and for whom address details were available. Almost all (97%, 353/363) of the sentinel cases with pH1N1 infection identified in 2009 surveillance were identified during this period. These individuals were contacted by mail. Each potential participant was sent patient information, a consent form and study questionnaire. Participants were asked to return the consent form and questionnaire by mail. Non-respondents were sent a second letter four weeks after the initial mailing. Questionnaire data were only used if the relevant consent form was received.

### Data collection

The questionnaire content was informed by early findings on the clinical spectrum of pH1N1, potential risk factors and known characteristics of seasonal influenza. The questionnaire collected demographic details, height and weight, illness characteristics, household contact details, exposure history, vaccination history, and co-morbidities (including pregnancy). Duration of symptoms was defined as the number of days between the index case's first and last symptoms. These symptoms could be any reported during the illness (not only fever, cough and fatigue). We recorded the age of the index case (in years) but the age of contacts was only categorised as child (less than 18 years old) or adult. Information on household contacts was provided by the index case (or parent/guardian if the index case was a child) and ILI in a contact was defined as ‘fever, cough, tiredness’.

The government's response to the pandemic was based on phases recommended in the Australian Health Management Plan for Pandemic Influenza. [20] The first Delay phase was implemented throughout Australia on 28^th^ April 2009. During the Delay, Contain and Modified Sustain phases, antiviral treatment with oseltamivir or zanamivir was recommended for all confirmed cases of pH1N1. [21] During these phases antiviral therapy was also recommended for all suspected cases and the close contacts of suspected and confirmed cases. A suspected case was defined as an individual with fever and a recent onset of rhinorrhoea, nasal congestion, sore throat or cough, and either close contact with a confirmed case within the last seven days or recent travel to a country with known local transmission. From the 23^rd^ June 2009, when Victoria moved to the Protect phase, antiviral medication was no longer recommended for cases with mild infection or for household contacts. Rates of antiviral treatment were compared across pandemic phases to examine the impact of phase recommendations on treatment patterns.

Key aspects of the Victorian Government's Pandemic Plan included isolation of confirmed and suspected cases and voluntary home quarantine for household contacts of suspected and confirmed cases. During the Delay and Contain phase confirmed and suspected cases were advised by the state Department of Health to isolate themselves for seven days following the commencement of antiviral treatment. During these phases the household contacts of both confirmed and suspected cases were also requested by the Department of Health, and through advice delivered by treating doctors, to remain in voluntary home quarantine for seven days, although there was no mechanism to enforce this advice or determine whether it had been followed. The requirement for household contact quarantine was lifted during the Modified sustain phase. In this phase confirmed cases were only asked to isolate themselves for three days following commencement of antiviral treatment. The study questionnaire asked if the index case or any member of the household was voluntarily isolated or quarantined. Participants were not asked to specify whether the household contacts who undertook voluntary quarantine were also contacts who developed influenza-like symptoms. We did not gather information on whether the entire household complied with the voluntary quarantine and from whom advice about voluntary quarantine had been received.

For household contact data, the index case was defined as the participant who had been identified through sentinel surveillance and who was the focus of the questionnaire. Household contacts were any other people living in the household. The index case was asked to record the number of household contacts above and below 18 years of age, the number who developed influenza-like symptoms within one week of the index case becoming ill, the age of unwell contacts, their relationship to the index case, and whether the contact received antiviral treatment. No laboratory testing was done for household contacts.

### Statistical analysis

Categorical variables were compared using Pearson's χ^2^ and Fisher's exact tests. Nonparametric data were compared using Wilcoxon rank sum tests, with p values <0.05 considered statistically significant. Crude secondary attack rates were calculated according to household size by dividing the number of contacts who became unwell by the total number of contacts in households of that size.

To determine whether there were associations between particular index case characteristics and ILI in a contact, univariate and multivariate logistic regression was conducted. Generalised estimating equations (GEE) were used to account for household clustering in the logistic regression model. Factors significant at *P* = 0.10 in univariate analysis were included in multivariate analysis. Possible risk factors included whether the index case was a child or adult, the number of children and adults in the household, whether contacts voluntarily quarantined themselves, whether the index case received antiviral treatment, the index case symptoms (e.g. cough) and symptom duration. The number of children in the household was included in the model as children have previously been found to be more infectious and susceptible to influenza infection than adults. [17], [22], [23] Statistical analysis was performed using Stata/IC 10.0 for Windows.

## Results

### Patient and illness characteristics

In total, 953 patients had nose and throat swabs taken at a sentinel GP practice between 1^st^ May and 31^st^ August 2009, when this study commenced. Of these, 353 (37%) tested positive for pH1N1 and were sent a study questionnaire. Completed questionnaires were returned by 132 patients and eleven letters were ‘returned to sender’ due to incorrect address details. The response rate was 132/342 (39%) of potential participants who received a letter.

Study participants were similar to all positive surveillance cases in terms of gender and median age (Table 1). However there were some differences in important age categories, with a greater proportion of study participants aged 10–17 years old (23%; 31/132) compared to all surveillance patients (15%; 54/353) (p = 0.04). The study included fewer participants who were 18–49 years old (46%; 61/132) compared with all surveillance patients (62%, 219/353) (p = 0.002). Thirty-seven cases did not provide information about Aboriginal or Torres Strait Islander status while all other cases reported no Indigenous background. Indigenous status was not considered in our analysis.

**Table 1:** Demographic characteristics of the 132 study respondents compared with sentinel surveillance cases and the Victorian population.

		Victorian Population (%) (n = 5,313,823)	Surveillance Cases (%) (n = 353)	Case Series Participants (%) (n = 132)
Gender				
	Female	50	173 (49)	75 (57)
Median Age (range)		37	22 (10 mths –78 yrs)	21 (10 mths –64 yrs)
Age Group				
	0–23 months	3	2 (1)	1 (1)
	2–4 years	4	10 (3)	3 (2)
	5–9 years	6	37 (10)	19 (14)
	10–17 years	10	54 (15)	31 (23)†
	18–49 years	46	219 (62)	61 (46)†
	50–64 years	18	30 (8)	17 (13)
	≥65 years	14	1 (0)	0 (0)

† p<0.05 on univariate analysis of case series versus surveillance cases.

Of the 132 cases, 28 (21%) had underlying medical conditions, including 11% of children (younger than 18 years) and 28% of adults (Table 2). Of 75 adults, seven (9%) were obese and five (7%) were morbidly obese. Two (4%) of 47 children were obese. Two of the obese adults and one of the obese children had an underlying medical condition. Two (40%) morbidly obese adults had underlying medical conditions. A total of 28 cases (21%) reported receiving the 2009 seasonal influenza vaccine, 15% of children and 26% of adults. Eight study participants reported working as health care workers while 76 (57%) were school or university students.

**Table 2:** Underlying medical conditions, body mass index and illness characteristics of index cases, according to age group.

Clinical Characteristics		All Participants Total (%) (n = 132)	Children <18 years Total (%) (n = 54)	Adults ≥18 years Total (%) (n = 78)
Underlying Medical Conditions				
	Any one condition	28 (21)	6 (11)	22 (28)
	Pregnancy	1 (1)	0 (0)	1 (1)
	Hypertension	10 (8)	0 (0)	10 (13)*
	Chronic respiratory disease	7 (5)	1 (2)	6 (8)
	Immunocompromised	2 (2)	1 (2)	1 (1)
	Obstructive sleep apnoea	1 (1)	0 (0)	1 (1)
	Cardiac disease	3 (2)	1 (2)	2 (3)
	Neurological disease	1 (1)	0 (0)	1 (1)
	Type I Diabetes	1 (1)	0 (0)	1 (1)
	Type II Diabetes	1 (1)	0 (0)	1 (1)
	Haematological disease	2 (2)	1 (2)	1 (1)
	Renal disease	0 (0)	0 (0)	0 (0)
	Other chronic disease	7 (5)	3 (6)	4 (5)
Obese (BMI 30–34.9 in adults ≥18 years or BMI percentile 95–100 in children aged 2 to 18 years) 1		9 (7)	2 (4)	7 (9)
Morbidly obese (BMI ≥35 in adults only) 1		5 (4)	-	5 (7)

Illness Characteristics				
Median days from symptom onset to swab (IQR) 2		1 (IQR 0, 3)	1 (IQR 0, 3)	1 (IQR 0, 3)
Median duration of symptoms (days) 3		7 (IQR 5, 11)	6.5 (IQR 4, 10)	9 (IQR 5, 14)**
Self-reported clinical symptoms				
	Cough	86 (65)	33 (61)	53 (68)
	Fever	123 (93)	52 (96)	71 (91)
	Shakes/chills	88 (67)	35 (65)	53 (68)
	Sore throat	82 (62)	32 (59)	50 (64)
	Shortness of breath	38 (29)	12 (22)	26 (33)
	Rhinorrhoea	53 (40)	27 (50)	26 (33)
	Blocked nose	43 (33)	14 (26)	29 (37)
	Body aches	104 (79)	38 (69)	66 (86)*
	Diarrhoea	14 (11)	6 (11)	8 (10)
	Vomiting	23 (17)	12 (22)	11 (14)
	Nausea	37 (28)	13 (24)	24 (31)
	Sore, red eyes	17 (13)	10 (19)	7 (9)
	Myalgia	78 (59)	24 (44)	54 (69) ***
	Arthralgia	51 (39)	22 (41)	29 (37)
	Fatigue	122 (92)	52 (96)	70 (90)

IQR  =  interquartile range.

*p<0.05 on univariate analysis of children versus adult cases.

**p<0.05 on Wilcoxon ranksum analysis.

***p<0.01 on univariate analysis of children versus adult cases.

1 Body Mass Index (BMI, weight in kilograms divided by height squared in metres) Excludes one pregnant women, one child under two, and 8 patients (6 children and 2 adults) missing height or weight data.

2 Excludes one child and 6 adults with missing data.

3 Excludes eight adults with missing data.

The most common self-reported symptoms were fever (93%), fatigue (92%) and ‘body aches’ (79%); 71% (94/132) of cases reported all three symptoms while 58% (76/132) reported the triad of fever, cough and fatigue. Despite fever forming part of the ILI case definition, review of symptom details provided by GPs with the surveillance swabs showed that some GPs tested patients who did not present with fever but in whom they nonetheless suspected influenza. Significantly more adults than children reported myalgia and ‘body aches’. Fourteen (11%) cases reported diarrhoea and 23 (17%) reported vomiting. The median duration of symptoms reported by adults was significantly longer (9 days) than that reported for children (7 days, p = 0.003).

### Treatment

About half the index cases (48%; 63/130), both adults and children, received antiviral treatment. Almost all index cases (95%; 59/62) who received antivirals reported completing the full treatment course. The median duration of symptoms in children who received antivirals was five days (interquartile range (IQR) 4, 7) compared with seven days (IQR 5.5, 10) in those who did not (p = 0.03). However no difference was seen among adults; those who received antivirals reported a median symptom duration of ten days (IQR 5, 14) compared with nine days (IQR 7, 12) in those who did not (p = 0.68).

There was no significant reduction in the proportion of index cases receiving antiviral treatment in the Protect phase, with 42/89 (47%) of the cases diagnosed during this phase receiving antivirals compared with 21/41 (51%) of those diagnosed in earlier pandemic phases (p = 0.67). However, 18/77 (23%) of the contacts of cases diagnosed during the Protect phase compared with 17/38 (45%) of contacts of cases diagnosed prior to the Protect phase reported receiving antivirals (p = 0.02).

Five participants, two children and three adults, reported hospitalisation. A hospitalised child reported a one day admission, and two adult cases reported a one day and six day admission. Duration was not reported for the other two cases.

Of 131 cases who answered questions regarding isolation during their illness, 67 (51%) reported isolating themselves. The median duration of isolation was five (IQR 3, 7) days for adults and seven (IQR 4, 7) days for children (p = 0.10). Of the 123 cases who lived with others, 23 (18%) reported other household members undergoing voluntary home quarantine. The duration of voluntary household quarantine was longer if the index case was a child (median 7 days; IQR 4, 7) than if the index case was an adult (median 3 days; IQR 2.5, 5) (p<0.01).

### Household Contacts

A total of 122 of 132 index cases lived in households with between two and nine household members. One lived in a university college with 250 ‘household’ members and was excluded from the household analysis. Eight cases lived alone and one case did not provide household details.

The 122 index cases had a total of 351 household contacts, of whom 115 were reported as developing influenza-like symptoms within one week of the index case, a crude household secondary attack rate of 33%. The median age of contacts with reported ILI was 24 years (range 1 to 69 years). There was no significant difference in the median ages of index cases and unwell contacts (p = 0.26).

Assumed influenza transmission was observed in 63 (51%) households. Of these, index cases in 32 (51%) households reported one secondary case, 19 (15%) reported two, five (8%) reported three, and seven (11%) reported four or more secondary cases. The crude secondary attack rate did not alter significantly according to household size (Table 3).

**Table 3:** Number of unwell household contacts and crude secondary attack rate (%) by household size.

Number of household members (including index case) within household	Number of households (n = 122)	Number of unwell household contacts (not including index case)							
		0 (%)	1	2	3	4	5	Total number of contacts with ILI	Crude secondary attack rate
2	24	15 (63)	9	-	-	-	-	9	38%
3	20	12 (60)	5	3	-	-	-	11	28%
4	42	20 (48)	7	10	5	-	-	42	33%
5	25	9 (36)	9	4	-	3	-	29	29%
6	7	3 (43)	-	2	-	-	2	14	40%
7	3	1 (33)	-	1	-	1	-	6	33%
8	0	-	-	-	-	-	-	-	-
9	1	0 (0)	0	0	0	1	0	4	50%

We determined if an association existed between particular index case characteristics and the occurrence of ILI in household contacts (Table 4). After controlling for clustering of contacts within households, contacts of index cases with cough (odds ratio (OR) 2.25, 95% CI 1.08, 4.69) and diarrhoea (OR 3.22, 95% CI 1.15, 9.03) were found to be significantly more likely to become unwell than contacts of index cases without these symptoms. Contacts of index cases with symptom duration greater than one week were also significantly more likely to become unwell (OR 3.06, 95% CI 1.57, 5.98). Quarantining of the household was significantly associated with an increased risk of household contacts developing ILI (OR 2.76, 95% CI 1.23, 6.21). Whether the index case was a child or an adult did not affect the risk of a household contact developing an ILI. The risk increased with the number of children in the household (OR = 1.37, 95% CI 1.11, 1.69) but not the number of adults.

**Table 4:** Risk factors for transmission of influenza A (H1N1) from index case to household contacts.

		Contacts (n = 351)			
Characteristic of index case	Number (%) of homes (n = 122)	Well Contacts (n = 236)	Unwell Contacts (n = 115)	Univariate odds ratio (95% CI)	Multivariate odds ratio (95% CI)
Age of index caseChild (0–17 years)Adult (≥18 years)	53 (43)69 (57)	13898	5956	0.83 (0.45, 1.51)1	
Index case symptomCoughRunny noseDiarrhoeaVomiting	82 (67)50 (41)14 (11)22 (18)	141 (60)95 (40)19 (8)45 (19)	90 (78)49 (43)21 (18)28 (24)	2.60 (1.36, 4.98)1.13 (0.62, 2.04)2.47 (0.97, 6.28)1.48 (0.72, 3.03)	2.25 (1.08, 4.69)*3.22 (1.15, 9.03)*
Symptom length (n = 337)>1 week≤1 week	60(49)62 (51)	152 (66)79 (34)	49 (46)57 (54)	2.34 (1.27, 4.32)1	3.06 (1.57, 5.98)**
Antiviral treatment (n = 345)	59 (48)	106 (45)	59 (52)	1.45 (0.79, 2.67)	
Isolation/quarantineIndex caseWhole family	61 (50)25 (20)	112 (47)31 (13)	58 (50)29 (25)	1.16 (0.63, 2.11)2.28 (1.01, 5.16)	2.76 (1.23, 6.21)*
HouseholdNumber of childrenNumber of adults				1.02 (0.82, 1.27)1.09 (0.91, 1.30)0.87 (0.55, 1.36)	1.37 (1.11, 1.69)**

CI  =  confidence interval.

*p<0.05 on univariate analysis of well and unwell contacts.

**p<0.01 on univariate analysis of well and unwell contacts.

## Discussion

We report a case series of community patients with pH1N1 during the peak of the 2009 Victorian influenza pandemic. The response rate of 39% was lower than expected and may reflect increasing community perceptions that the pandemic was not as severe as initially reported. Compared to all sentinel general practice patients with pandemic influenza, a higher proportion of 10–17 year olds and lower proportion of 18–49 year olds responded. In 2009, with significant concern about the spread of pH1N1 in schools and universities, 33% of sentinel surveillance swabs were collected at one of two university health clinics, suggesting that university students may have been over-represented amongst overall surveillance cases. A number of the ‘return to sender’ letters were from patients who had a positive swab taken at one of these clinics. This is likely to have contributed to a lower response rate in this group. The address details for all cases who agreed to participate were compared to ensure that only one response was received from any one household.

Previous work has shown infection with both seasonal and pH1N1 tends to occur in younger patients than influenza A (H3N2) and influenza B infections. [24], [25] The case median age of 21 years is similar to surveillance findings overseas,[26], [27], [28] suggesting greater susceptibility in younger age groups or potential sampling bias in younger surveillance patients. Medical co-morbidities were not common in index cases. This is perhaps not surprising given the study's community focus. Previous studies that identified high rates of underlying health problems have primarily focused on hospitalised patients. [5], [6], [28], [29], [30]

The clinical features reported by index cases were largely similar to known features of seasonal influenza. [31] A triad of influenza-like symptoms (fever, cough, and fatigue or malaise) was used to identify cases for swab collection in the surveillance program. Predictably these symptoms were frequently reported. Whilst diarrhoea or vomiting have been reported in as many as 39% of hospitalised patients with pH1N1 [6], [26], [32], in our study they were reported by only 11% and 17% of index cases respectively. A similarly low level of gastrointestinal symptoms was reported in a review of hospitalised patients in Victoria. [1]

All index cases had confirmed pH1N1 infection, but despite formal recommendations for all confirmed cases to receive antivirals in the early stages of the pandemic, only half the cases received antiviral medication and there was no difference in the rate of prescription before and after the Protect phase was implemented, although there was a significant reduction in the number of contacts receiving antivirals during the Protect phase, consistent with the recommended change in policy.

Controlled trials of antiviral treatment in uncomplicated seasonal influenza have reported reductions of approximately one day in illness duration and reduced illness severity. [2], [33] Observational studies have suggested that antivirals may reduce severity and disease mortality in hospitalised patients with pH1N1. [6], [34] Our finding of a significant difference in the median duration of symptoms in child index cases who received antivirals supports the observation that early neuraminidase inhibitor treatment may limit illness duration.

Our study also investigated transmission of pH1N1 in households. Our crude secondary attack rate calculation for ILI of 33% was comparable to secondary attack rates reported for laboratory-confirmed seasonal influenza [35], [36] and also similar to the secondary household attack rate for laboratory confirmed pH1N1 of 26% reported in Kenya. [29] However it is higher than the ILI rate of 10% reported in a large study of US household transmission and the ILI rate of 6% reported in a recent prospective Hong Kong household study, although in this study all contacts received hand hygiene advice and alcohol handrub. [15], [37]

We can suggest a number of reasons for an apparent higher secondary attack rate in our study than has been reported elsewhere. We did not confirm influenza in household contacts and it is possible that some contacts with ILI may have been infected with another respiratory virus. However nucleotide sequencing indicates that cases of influenza in a household in which an index case has a recently diagnosed seasonal influenza A are most often due to secondary household transmission rather than transmission from community sources. [38] Alternatively infection in a contact, if due to influenza, may have resulted from a contact outside the home. We were not able to exclude this possibility. Neither could we exclude the possibility of contemporaneous index cases in large households. We assumed the sentinel patient was the index case but it is possible that the sentinel case was a contact. These limitations may have resulted in an over estimate of presumed influenza transmission in household contacts.

However one highly plausible reason for the increased secondary attack rate observed in this study was due to voluntary isolation of cases and quarantine of household contacts. Where quarantine of an entire family occurred, we found the risk of reported secondary attack increased more than two and a half times. It is conceivable that families of more severely unwell, and thus potentially more infectious, index cases may have felt more compelled to voluntarily quarantine themselves.

Symptoms of cough and diarrhoea were associated with increased risk of household contacts becoming unwell. Cough is a known facilitator of droplet spread of influenza. [39], [40] There is little evidence of faecal-oral transmission of pH1N1, although direct or indirect contact through fomites has been considered a potential source of human-to-human transmission in avian H5N1 influenza. [41], [42] Some studies have suggested that high rates of gastrointestinal symptoms in hospitalised pH1N1 patients may be associated with more severe infection. [7] If diarrhoea is a marker of severity, those with diarrhoea and more severe infection (or greater viral load) may be more likely to transmit to household contacts. Contacts of adult index cases with symptom duration greater than one week were significantly more likely to develop ILI. Longer symptom duration may also represent both a more severe illness with a greater viral load, or a longer period of viral shedding.

Studies of seasonal influenza have found increased risk for contacts exposed to unwell preschool (0–5 years) or school-aged (6–15 years) children. [17], [23] We found the age category of the index case (child/adult) did not affect the risk of secondary transmission but that the risk increased with the number of children, but not the number of adults, in the household. These observations may imply that children were more susceptible contacts than adults, but not more efficient transmitters.

Index cases were detected by routine surveillance and were therefore subject to case-ascertainment bias, but secondary household cases should provide a less biased sample of illness severity. The questionnaire relied upon cases recalling details of their illness, leading to potential recall bias. The potential omission of more severe cases who may have been less likely to complete the questionnaire, clearly influences the representativeness of our findings. However three of 132 respondents (2%) reported being admitted to hospital. This was higher than the modeled rate (0.3%) reported for Victoria [43], suggesting relative severity may have been captured in our case series.

For most Victorians the 2009 influenza A (H1N1) pandemic was less severe than initially feared. Whilst a small proportion of cases required hospitalisation, the majority were managed in the community. It is likely that the greatest political and social impact of the virus came from efforts to manage the pandemic, not from the impact of the pandemic itself. Pandemic plans will need to be revised to allow a response commensurate with the risk. [44]
